# Cannabidivarin and cannabigerol induce unfolded protein response and angiogenesis dysregulation in placental trophoblast HTR-8/SVneo cells

**DOI:** 10.1007/s00204-024-03781-8

**Published:** 2024-05-15

**Authors:** Patrícia Alves, Cristina Amaral, Marina S. Gonçalves, Natércia Teixeira, Georgina Correia-da-Silva

**Affiliations:** 1 Faculty of Pharmacy, Laboratory of Biochemistry, UCIBIO, Applied Molecular Biosciences Unit, University of Porto, Rua Jorge de Viterbo Ferreira 228, 4050-313 Porto, Portugal; 2 Associate Laboratory i4HB, Institute for Health and Bioeconomy, University of Porto, Rua Jorge de Viterbo Ferreira, 228, 4050-313 Porto, Portugal; 3 Faculty of Pharmacy, Laboratory of Biochemistry, REQUIMTE, University of Porto, Rua Jorge de Viterbo Ferreira 228, 4050-313 Porto, Portugal; 4 Faculty of Pharmacy, University of Porto, Rua Jorge de Viterbo Ferreira 228, 4050-313 Porto, Portugal

**Keywords:** Cannabinoids, Cannabidivarin, Cannabigerol, Placenta, Endoplasmic reticulum stress, Angiogenesis

## Abstract

Cannabidivarin (CBDV) and cannabigerol (CBG) are minor phytocannabinoids from *Cannabis sativa*, whose health benefits have been reported. However, studies about the impact of these cannabinoids on fundamental cellular processes in placentation are scarce. Placental development involves physiological endoplasmic reticulum (ER) stress, however when exacerbated it can lead to altered angiogenesis and pregnancy disorders, such as intrauterine growth restriction and preeclampsia. In this work, the effects of CBDV and CBG (1–10 µM) on placental extravillous trophoblasts were studied, using the in vitro model HTR-8/SVneo cells. Both cannabinoids induced anti-proliferative effects and reactive oxygen/nitrogen species generation, which was dependent on transient receptor potential vanilloid 1 (TRPV1) activation. Moreover, CBDV and CBG significantly upregulated, in a TRPV-1 dependent manner, the gene expression of *HSPA5*/Glucose-regulated protein 78 (GRP78/BiP), a critical chaperone involved in ER stress and unfolded protein response (UPR) activation. Nevertheless, the UPR pathways were differentially activated. Both cannabinoids were able to recruit the IRE branch, while only CBDV enhanced the expression of downstream effectors of the PERK pathway, namely p-eIF2α, ATF4 and CHOP. It also augmented the activity of the apoptotic initiator caspases-8 and -9, though the effector caspases-3/-7 were not activated. TRB3 expression was increased by CBDV, which may hinder apoptosis termination. Moreover, both compounds upregulated the mRNA levels of the angiogenic factors *VEGFA*, *PGF* and *sFLT1*, and disrupted the endothelial-like behavior of HTR-8/SVneo cells, by reducing tube formation. Thus, CBDV and CBG treatment interferes with EVTs functions and may have a negative impact in placentation and in pregnancy outcome.

## Introduction

Cannabinoids from *Cannabis sativa*, mainly delta-9-tetrahydrocannabinol (THC) and cannabidiol (CBD), are a topic of deep interest in research due to their medicinal and recreational purposes (Thibaut and Hoehe 2020). Nevertheless, the so-called minor cannabinoids, present in smaller amounts in the plant, are also gaining pharmacological relevance (Franco et al. 2020). For cannabidivarin (CBDV), a propyl analogue of CBD, medicinal benefits have been explored in recent years, especially for the treatment of autism spectrum disorders and epilepsy (Zamberletti et al. 2021), while for cannabigerol (CBG) it is known that it presents appetite-stimulating properties (Anokwuru et al. 2022). Moreover, both compounds have antimicrobial, antioxidant and anti-inflammatory actions (Jastrząb et al. 2022; Pagano et al. 2019; Russo et al. 2021). In addition, they have anti-cancer potential, decreasing cell viability and proliferation in different cancer cell types (Tomko et al. 2020), as well as neuroprotective effects (Stone et al. 2021). However, little is known about the potential toxic effects of these cannabinoids on reproductive health. Our group has already unveiled the impact of the major phytocannabinoids CBD and THC in several processes that govern placental development and function (Alves et al. 2021, 2023; Costa et al. 2015; Maia et al. 2019), but the effects of the minor phytocannabinoids are unknown.

Placental development involves highly controlled processes of cell proliferation, differentiation, apoptosis, migration and invasion of trophoblast cells (Lunghi et al. 2007). Endoplasmic reticulum (ER) stress is crucial for placental development, and is involved in angiogenesis, cell proliferation, autophagy and apoptosis (Bastida-Ruiz et al. 2017). The ER is a quality control organelle that orchestrates the folding and post-translational maturation of proteins. An accumulation of misfolded or unfolded proteins generates ER stress and, to cope with this, a signaling response termed unfolded protein response (UPR) is activated, aiming to restore the homeostasis, through regulation of translation and promotion of the expression of specific genes, such as molecular chaperones, involved in protein folding or degradation (Bhattarai et al. 2021). This starts with the activation of transmembrane ER signaling proteins through dissociation of the chaperone Glucose-regulated protein 78 (GRP78/BiP), which binds to the misfolded proteins. Three different transmembrane sensor proteins are known, namely protein kinase R-like endoplasmic reticulum kinase (PERK), inositol-requiring enzyme 1 alpha (IRE1) and activating transcription factor 6 (ATF6). PERK activation increases the phosphorylation of the alpha subunit of the translation protein eIF2 (eIF2α), reducing protein translation to alleviate ER stress. Simultaneously, the phosphorylated eIF2α (p-eIF2α) induces the expression of the activating transcription factor 4 (ATF4), targeting the activation of genes to control cell fate, such as the pro-apoptotic gene *DDIT3* (C/EBP homologous protein CHOP) or autophagy-related genes. The activation of the RNase domain of IRE1 results in the production of the transcription factor spliced XBP1 (sXBP1), which is the active isoform of XBP1. This protein translocates to the nucleus increasing the expression of pro-survival genes, like chaperones. The third UPR sensor, ATF6, when activated translocates to the Golgi complex, where it is cleaved by specific proteases, and the resulting fragments induce the expression of chaperones and XBP1 (Tsang et al. 2010). However, when the ER stress is deregulated, the UPR is not able to tackle the increase of misfolded proteins. This may lead to a reduction of cell proliferation, alterations in angiogenesis and increase of apoptotic cell death, resulting in pregnancy disorders, such as intrauterine growth restriction (IUGR) and pre-eclampsia (Bastida-Ruiz et al. 2017; Burton et al. 2009). In fact, increased ER stress has been associated with impaired placental and fetal development (Iwawaki et al. 2009; Yung et al. 2008). In mice, loss of IRE1 led to a reduction in vascular endothelial growth factor-A (VEGF-A) and severe placental dysfunction (Iwawaki et al. 2009).

Extravillous trophoblast cells (EVTs) are one type of trophoblast cells of the placenta. They proliferate from anchoring chorionic villi and invade the decidualized endometrium and the uterine spiral arteries (Lunghi et al. 2007). As partakers in the angiogenesis and vessel remodeling processes that occur in placentation, EVTs express pro-angiogenic factors, such as VEGF-A and placental growth factor (PlGF), and their respective receptors, like VEGFR1 or fms-like tyrosine kinase-1 (Flt-1), VEGFR2, and VEGFR3, as well as the anti-angiogenic soluble form of Flt-1 (sFlt-1), which hinders VEGF and PlGF actions. A delicate equilibrium between pro-angiogenic and anti-angiogenic factors is required for EVTs function and adequate placentation (Charnock-Jones et al. 2004; Schiessl et al. 2009).

Considering that epidemiological studies have highlighted an increase of cannabis use by pregnant women (Volkow et al. 2019; Young-Wolff et al. 2019) and to shed light on the impact of cannabinoids in placentation, the aim of this work was to evaluate the effects of two minor phytocannabinoids with pharmacological interest, CBDV and CBG, on EVTs fundamental cellular processes, focusing on the relevance of ER stress and angiogenic properties, using the HTR-8/SVneo cell line, a well-accepted cell model of EVTs (Graham et al. 1993).

## Material and methods

### Cell culture

HTR-8/SVneo cell line (ATCC, Manassas, VA, USA) was maintained at 37 °C in a 5% CO_2_ humidified atmosphere with RPMI 1640 medium (Gibco/Invitrogen Corporation, Carlsbad, CA, USA). The cell culture medium was enriched with 10% fetal bovine serum (FBS) (PAN-Biotech, Aidenbach, Germany), 1% antibiotic–antimycotic solution (penicillin G, streptomycin, and amphotericin B, AB-AM) (PAN-Biotech, Aidenbach, Germany), 1.25% glucose (Gibco/Invitrogen Corporation, Carlsbad, CA, USA) and 1% sodium pyruvate (PAN-Biotech, Aidenbach, Germany). After adhesion, cells were exposed to different concentrations (1–10 µM) of CBDV and CBG (THC Pharm GmbH, Frankfurt, Germany) pre-diluted in cell culture medium with (1%) or without FBS for different time incubations (6, 24, or 48 h). The CBDV and CBG stock solutions, with a purity of 98.9% and 99.8%, respectively, were prepared in 100% dimethyl sulfoxide (DMSO, Sigma-Aldrich Co, Saint Louis, MO, USA) and stored at −20 °C. The final concentration of DMSO in the cell culture medium was below 0.05% for all experiments. DMSO at 0.05% was used in the control and per se had no impact on the viability of the HTR-8/SVneo cells.

### Cell viability assays

HTR-8/SVneo cells were seeded in 96-well plates at a cellular density of 5 × 10^3^ cells/well and exposed to CBDV and CBG (1–10 µM) for 24 and 48 h. After the incubation time, MTT (M5655, Sigma-Aldrich Co, Saint Louis, MO, USA) was added at a final concentration of 0.5 mg/mL and incubated for 3 h at 37 °C. The resulting purple formazan was subsequently dissolved in a solution of DMSO and isopropanol (3:1). Absorbance was measured at 540 nm, using a Biotek Synergy HTX Multi-Mode Microplate Reader (Biotek Instruments, Vermont, USA).

The activity of the cytoplasmic enzyme lactate dehydrogenase (LDH), released into the culture medium in case of cell necrosis, was evaluated using the CytoTox 96® non-radioactive cytotoxicity assay kit (G1781, Promega, Madison, WI, USA), following the manufacturer's provided guidelines.

### Cell cycle analysis

HTR-8/SVneo cells were seeded in 6-well plates (4 × 10^5^ cells/well) and exposed to CBDV and CBG (5 μM) for 48 h. As previously described (Alves et al. 2021), after this, cells were collected and fixed with cold ethanol 70%. Then, cells were treated with a DNA staining solution containing 5 μg/mL propidium iodide (PI) (P-4170, Sigma-Aldrich Co., Saint Louis, USA), 0.1% Triton X-100 (Sigma-Aldrich Co., Saint Louis, USA) and 200 μg/mL Dnase-free Rnase A (GE011.0100, GriSP Research Solutions, Porto, Portugal) diluted in PBS. DNA content was measured through flow cytometry in a BD Accuri™ C6 cytometer (San Jose, CA, USA). The cytometer, equipped with BD Accuri™ C6 software, featured detectors for three fluorescence channels (FL-1, FL-2, FL-3) and for forward (FSC) and side (SSC) light scatter channels set on a linear scale. Data was collected from 40 000 events/cells, and gates were applied to exclude debris, cell doublets, and aggregates. Singlet cells were analyzed using a two-parameter plot of FL-2-Area to FL-2-Width for PI fluorescence. The acquired data were processed using BD Accuri™ C6 software. Results are presented as percentage of total cells in the G_0_/G_1_, S and G_2_/M cell cycle phases.

### RT‑PCR analysis

HTR-8/SVneo cells were seeded in 6-well plates (4 × 10^5^ cells/well) and treated with CBDV and CBG at 5 µM, with or without the transient receptor potential vanilloid 1 (TRPV1) antagonist capsazepine (CPZ, 0.2 µM, 0464, Tocris Bioscience, Bristol, UK), the IRE1 inhibitor 4µ8c (1 µM, SML0949, Sigma-Aldrich Co, Saint Louis, MO, USA) or the selective inhibitor of PERK, GSK 2656157 (GSK, 0.5 µM, sc-490341, Santa Cruz Biotechnology, CA, USA), which were added 30 min before the co-incubation with cannabinoids. The ER stress inducer thapsigargin (TG, 0.1 µM, sc-24017, Santa Cruz Biotechnology, CA, USA) was used as positive control. After 48 h, cells were collected in TripleXtractor (GB23.0200, GRiSP, Porto, Portugal) and RNA was extracted, according to manufacturer’s instructions. RNA quantification was performed in the NanoDrop ND-1000 Spectrophotometer (NanoDrop Technologies, Inc, Wilmington, DE, USA). cDNA was obtained through reverse transcription of RNA with the Xpert cDNA Synthesis Supermix (GK86.0100, GRiSP, Porto, Portugal). Amplification was achieved using Xpert Fast SYBR (GE20.2501, GRiSP, Porto, Portugal) and specific primers in the Applied Biosystems StepOnePlus™ Real-Time PCR system. Table 1 displays the primer sequences and qRT-PCR conditions. Gene expression was normalized using two housekeeping genes, *ACTB* (β-actin) and *TUBA1A* (α-tubulin) and analysis was carried out using 2^−ΔΔCt^ method, with *ACTB* as reference gene. Results are presented as fold change in gene expression, in comparison with the control.

**Table 1:** Primer sequences and RT-PCR conditions used to assess the gene expression of *HSPA5*, *DDIT3*, *ATF4*, *sXBP1*, *VEGFA*, *PGF*, *FLT1* and *sFLT1*. *ACTB and TUBA1A* were used as housekeeping controls

Gene Symbol	Primer sequence (5′-3′)	Annealing temperature (^°^C)	Reference
*HSPA5*	Sense: TTCTGCTGTATCCTCTTCACCAGT Anti-sense: TGTTCAACCAATTATCAGCAAACTC	61.1	Almada et al. 2020
*DDIT3*	Sense: TCTCCTTCATGCGCTGCTTT Anti-sense: AGAACCAGGAAACGGAAACAGA	57.0	Almada et al. 2020
*ATF4*	Sense: ATCCTGCTTGCTGTTGTTGG Anti-sense: GTTCTCCAGCGACAAGGCTA	61.1	Almada et al. 2020
*sXBP1*	Sense: ATCCATGGGGAGATGTTCTGG Anti-sense: CTGAGTCCGAATCAGGTGCAG	60.0	Ferreira et al. 2022
*VEGFA*	Sense: TGCATTCACATTTGTTGTGCTGTAG Anti-sense: TGCAGATTATGCGGATCAAACC	57.0	Maia et al. 2022
*PGF*	Sense: GTCTCTCTCCTCCAAGGGGT Anti-sense: GAGACCCACAGACTGCCAC	58.0	Maia et al. 2022
*FLT1*	Sense: TTCCAGCTCAGCGTGGTCGTA Anti-sense: CAGGCCCAGTTTCTGCCATT	60.0	Maia et al. 2022
*sFLT1*	Sense: ACAATCAGAGGTGAGCACTGCAA Anti-sense: TCCGAGCCTGAAAGTTAGCAA	62.0	Maia et al. 2022
*ACTB*	Sense: TGCCATCCTAAAAGCCACCC Anti-sense: AGACCAAAAGCCTTCATACATCTC	55.0	Sousa et al. 2022
*TUBA1A*	Sense: CTGGAGCACTCTGATTGT Anti-sense: ATAAGGCGGTTAAGGTTAGT	55.0	Amaral et al. 2021

### Western blotting

Like previously described (Alves et al. 2021), HTR-8/SVneo cells (4 × 10^5^/well) were seeded in 6-well plates and treated with CBDV and CBG (5 µM), with or without CPZ (0.2 µM), for 48 h. Protein samples (25 µg for most proteins and 50 µg for CHOP and tribbles-related protein 3, TRB3) were separated by 10 or 12% SDS–PAGE and transferred onto nitrocellulose membranes. Membranes were incubated with mouse monoclonal antibodies against CHOP (1:100, sc-7351; Santa Cruz Biotechnology, CA, USA) and TRB3 (1:100, sc-271572; Santa Cruz Biotechnology, CA, USA), or rabbit monoclonal or polyclonal antibodies against p-eIF2α (1:200, 3398S; Cell Signaling Technology, Leiden, The Netherlands), eIF2α (1:200, 5342S; Cell Signaling Technology, Leiden, The Netherlands), caspase-3 (1:200, sc-7148; Santa Cruz Biotechnology, CA, USA), poly (ADP-ribose) polymerase-1 (PARP-1) (1:200, 9542S; Cell Signaling Technology, Leiden, The Netherlands), p-AKT (Ser473) (1:200, 4060S; Cell Signaling Technology, Leiden, The Netherlands) and AKT (1:200, 4691S; Cell Signaling Technology, Leiden, The Netherlands) at 4 °C overnight. Then, membranes were washed and incubated with peroxidase-conjugated secondary antibody anti-rabbit or anti-mouse (1:1000 or 1:2000; Thermo Fisher, Waltham, MA, USA). Immunoreactive bands were visualized using a chemiluminescent substrate WesternBright™ ECL HRP substrate (K-12045-D20, Advansta, Menlo Park, USA) and a ChemiDoc™ Touch Imaging System (Bio-Rad, Laboratories Melville, NY, USA). Stripping was performed and the membranes were incubated with mouse monoclonal antibody against β-actin (1:500, sc-47778; Santa Cruz Biotechnology, CA, USA), used as loading control.

### Evaluation of mitochondrial transmembrane potential (Δψm) and intracellular reactive oxygen species (ROS) production

HTR-8/SVneo cells were seeded in 96-well black plates (5 × 10^3^ cells/well) and treated with CBDV and CBG (5 µM), with or without CPZ (0.2 µM), N-acetylcysteine (NAC, 1 mM, A0150000, Sigma-Aldrich Co, Saint Louis, MO, USA), 4µ8c (1 µM) or GSK (0.5 µM). Mitochondrial transmembrane potential (Δψm) was evaluated after 48 h of exposure, as previously described, using the fluorescent probe 3,3′-dihexyloxacarbocyanine iodide (DiOC_6_, 100 nM, D273, Thermo Fisher, Waltham, MA, USA) (Alves et al. 2021). The mitochondrial transmembrane depolarizing agent carbonyl cyanide m-chlorophenylhydrazone (CCCP, 30 µM, C2759, Sigma-Aldrich Co, Saint Louis, MO, USA) was used as a positive control. The generation of intracellular reactive oxygen species (ROS) was assessed by incubation with 2ʹ,7ʹ-dichlorodihydrofuoresceindiacetate (DCDHF-DA, 25 µM, D6883, Sigma-Aldrich Co, Saint Louis, MO, USA) for 1 h, as previously reported (Alves et al. 2021). The emitted fluorescence was measured at different time periods (0, 24 and 48 h). For positive control, cells were incubated with phorbol 12-myristate 13-acetate (PMA, 50 ng/mL, 79,346, Sigma-Aldrich Co, Saint Louis, MO, USA). Biotek Synergy HTX Multi-Mode Microplate Reader (Biotek Instruments, Vermont, USA) was used to measure fluorescence and the results were presented as relative fluorescence units (RFU).

### Determination of caspases‑3/‑7, -8 and ‑9 activities

HTR-8/SVneo cells were seeded in 96-well white plates (5 × 10^3^ cells/well) and incubated with CBDV and CBG at 5 μM for 48 h. In addition, CPZ (0.2 µM), NAC (1 mM), 4µ8c (1 µM) or GSK (0.5 µM) were also added to cells treated with or without cannabinoids. The activities of caspases-3/-7, -8 and -9 were assessed through a luminescence assay, using Caspase-Glo® 3/7 (G811C), Caspase-Glo® 8 (G815C) and Caspase-Glo® 9 (G816C) kits (Promega, Madison, WI, USA), according to manufacturer’s instructions. The resultant luminescence was measured in a Biotek Synergy HTX Multi-Mode Microplate Reader (Biotek Instruments, Vermont, USA). Staurosporine (STS, 10 μM, S4400, Sigma-Aldrich Co, Saint Louis, MO, USA), an inducer of apoptosis, was used as positive control. Results are expressed in relative luminescence units (RLU).

### Tube formation assay

The impact of CBDV and CBG in the tube formation of HTR-8/SVneo cells was evaluated at 2 μM. For this, growth factor reduced Matrigel (50 μL, 3,56,230, Corning, NY, USA) was added to 96-well plates and incubated for 30 min at 37 °C to solidify, as described previously (Maia et al. 2022). After this step, cells were seeded (1.5 × 10^4^ cells/well) and incubated with the compounds, in RPMI 1640 FBS-free medium, for 6 h. Images from three random different fields/well were acquired (× 100 magnification) under a phase contrast microscope (Eclipse 400, Nikon, Japan), using Nikon NIS Elements Software. Analysis was carried out using the Angiogenesis Analyzer plugin for Image J (Carpentier et al. 2020) and the results presented as total segment length (µm), in comparison with the control.

### Statistical analysis

Statistical analysis was carried out by ANOVA, followed by Bonferroni post hoc-test to make pairwise comparisons of individual means (GraphPad PRISM v.8.0, GraphPad Software, Inc., San Diego, CA, USA). At least three independent experiments were performed in triplicate. Data are expressed as the mean ± SEM and differences were statistically significant at *p* < 0.05.

## Results

### Anti-proliferative effects of cannabidivarin and cannabigerol

**Fig. 1:**
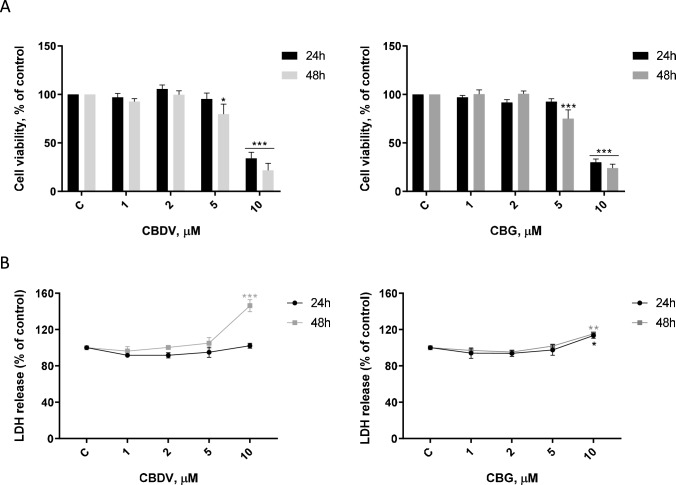
CBDV and CBG impact on cell viability. HTR-8/SVneo cells were treated with different concentrations (1–10 µM) of the compounds for 24 and 48 h. Effects were assessed through MTT assay **(A)** and LDH release **(B)**. Results are presented as mean ± SEM (n = 4). Significant differences between control and CBDV or CBG-treated cells are denoted by * (*p* < 0.05), ** (*p* < 0.01) and *** (*p* < 0.001)

The impact of CBDV and CBG at different concentrations (1–10 µM) on HTR-8/SVneo cells’ viability was evaluated through MTT and LDH assays after 24 and 48 h of treatment. Both CBDV and CBG decreased cell viability at 5 µM at 48 h (*p* < 0.05 and *p* < 0.001, respectively), and at 10 µM (*p* < 0.001) at both incubation periods (Fig. 1A). LDH release occurred only at 10 µM, after 48 h in the case of CBDV (*p* < 0.001), and at 24 (*p* < 0.05) and 48 h (*p* < 0.01) for CBG (Fig. 1B). To assess if the decrease on cell viability observed for both compounds at 5 µM was associated with anti-proliferative effects, their impact on cell cycle progression was evaluated. As showed in Table 2, CBDV and CBG induced a significant (*p* < 0.05, *p* < 0.01) arrest of cells at G_0_/G_1_ phase of 2.80% and 3.65% respectively, in comparison with the control. Moreover, a significant (*p* < 0.05, *p* < 0.01) reduction of S phase was observed for both cannabinoids (2.89% for CBDV and 3.37% for CBG, in relation to the control).

**Table 2:** Cell cycle distribution of HTR-8/SVneo cells exposed to CBDV and CBG

	**Control**	**CBD**V** 5**	**CBG 5**
G_0_/G_1_	61.92 ± 0.75	64.72 ± 0.95 *	65.57 ± 1.16 **
S	12.23 ± 0.40	9.34 ± 0.59 *	8.86 ± 0.32 **
G_2_/M	26.91 ± 0.69	26.16 ± 0.63	27.47 ± 1.02

HTR-8/SVneo cells were treated with CBDV and CBG (5 µM) for 48 h. DNA content was assessed through flow cytometry with PI labelling. Results are shown as single cell events in the G_0_/G_1_, S and G_2_/M phases of cell cycle. Data represents means ± SEM (*n* = 3). Significant differences between control and treated cells are indicated by * (*p* < 0.05) and ** (*p* < 0.01)

### Cannabidivarin and cannabigerol activate UPR through different signaling pathways

Given the role of ER stress in placental development (Bastida-Ruiz et al. 2017), the effects of CBDV and CBG on the expression of relevant ER markers, as well as the involvement of TRPV1 in this process were explored, since it seems to modulate ER stress (Vestuto et al. 2022). We also evaluated the response of each arm of the UPR to TG, a classical ER stressor.

**Fig. 2:**
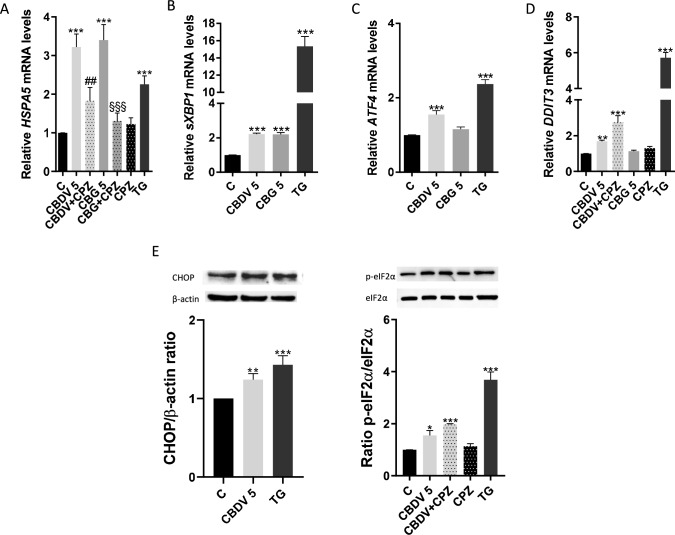
CBDV and CBG increase the expression of ER-stress markers. HTR-8/SVneo cells were treated with CBDV and CBG (5 μM) for 48 h, with or without the TRPV1 antagonist CPZ (0.2 µM). **A-D** Expression of the *HSPA5*, *sXBP1*, *ATF4* and *DDIT3* genes was analyzed through RT-PCR. Results show transcript levels normalized against *ACTB*. **E** The protein expression of p-eIF2α and CHOP was evaluated by Western blot. TG (0.1 µM), an ER-stress inducer, was used as positive control. Data are presented as the mean ± SEM (n = 5). Significant differences between control and CBDV or CBG-treated cells are denoted by * (*p* < 0.05), ** (*p* < 0.01) and *** (*p* < 0.001); between CBDV and CBDV + CPZ by ## (*p* < 0.01); between CBG and CBG + CPZ by §§§ (*p* < 0.001)

Both cannabinoids activated UPR, increasing the gene expression of chaperone *HSPA5* (*p* < 0.001), in a TRPV1-dependent manner (Fig. 2A), as the increase of *HSPA5* was significantly reversed (*p* < 0.01, *p* < 0.001) by CPZ. IRE1 pathway was activated by both CBDV and CBG, which augmented the expression of the *sXBP1* gene (*p* < 0.001) (Fig. 2B). Only CBDV was able to recruit the PERK signaling pathway, with increased expression of the mRNA levels of *ATF4* (*p* < 0.001) and *DDIT3* (*p* < 0.01)*,* being the latter a TRPV-1 independent effect (Fig. 2C, D). Moreover, these actions were confirmed through the increase of CHOP expression (*p* < 0.01) and of the phosphorylated form of eIF2α (*p* < 0.05), which was also independent of TRPV1 activation (Fig. 2E). As expected, the ER stress inducer TG significantly (*p* < 0.001) increased the expression of these ER markers.

### Evaluation of apoptosis, reactive oxygen/nitrogen species (ROS/RNS) production and mitochondrial transmembrane potential (Δψm)

**Fig. 3:**
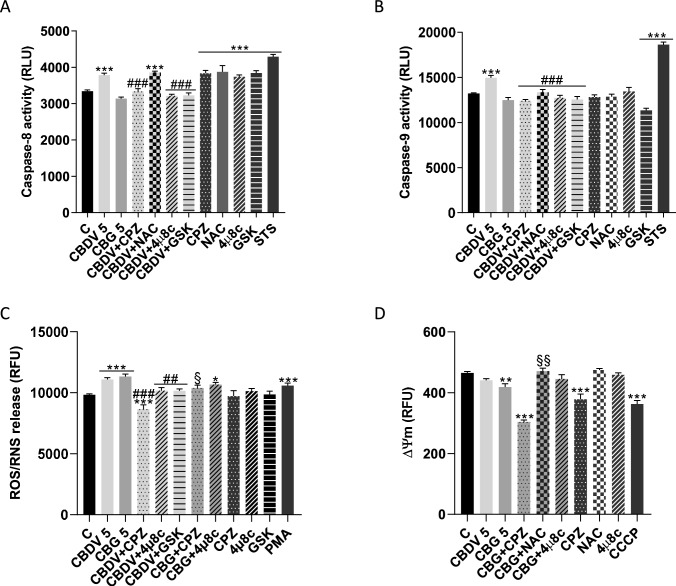
Effects of CBDV and CBG on caspase activation, ROS/RNS generation and mitochondrial membrane potential. **A, B** Evaluation of the activity of the initiator caspases-8 and -9 (48 h); **C** ROS/RNS generation (0 h) and **D** of Δψm (48 h). CBDV and CBG-treated cells (5 µM) were pre-incubated with CPZ (0.2 µM), 4µ8c (1 µM) or GSK (0.5 µM), to study the dependence of TRPV1 and UPR pathways. CCCP (30 µM) and PMA (50 ng/mL) were used as positive controls for Δψm and ROS generation, respectively. STS (10 µM) was used as inducer of apoptosis. The results are presented as mean ± SEM (n = 3). Significant differences between control and treatments are indicated by * (*p* < 0.05), ** (*p* < 0.01) and *** (*p* < 0.001); between CBDV-treated cells with or without CPZ, 4µ8c or GSK are denoted by ## (*p* < 0.01) and ### (*p* < 0.001); between CBG-treated cells with or without CPZ or 4µ8c are identified by § (*p* < 0.05) and §§ (*p* < 0.01)

PERK, IRE1 and ATF6 and their downstream cascades are involved in cell death induced by unresolved ER stress, being the axis PERK/ATF4/CHOP well-described as playing a crucial role. Cell death can occur by activation of the mitochondrial pathway. In addition, ER stress and proteotoxic stress have also been shown to induce the activation of caspase-8 (Iurlaro and Muñoz-Pinedo 2016; Szegezdi et al. 2006). Therefore, the effects of CBDV and CBG on the activity of the initiator caspases-8 and -9 were evaluated. Additionally, given the interaction between mitochondria, ER and oxidative stress (Burton et al. 2017), we assessed their ability to produce ROS/RNS and to reduce Δψm. Moreover, as CBDV and CBG are both agonists of TRPV1, which is involved in cell death (Fonseca et al. 2018), its role was explored through the co-incubation of cannabinoids with the antagonist CPZ. Contrary to CBG, CBDV induced the activation of the initiator caspases-8 and -9 (*p* < 0.001) after 48 h, through TRPV1 (*p* < 0.001) (Fig. 3A, B). In addition, both CBDV and CBG significantly (*p* < 0.001) increased ROS/RNS release via TRPV1 activation (*p* < 0.001; *p* < 0.05) (Fig. 3C). On the other hand, after 48 h of treatment, only CBG significantly (*p* < 0.01) decreased Δψm, in a TRPV1-independent manner (Fig. 3D). As ROS/RNS can induce caspases activation and mitochondrial dysfunction (Redza-Dutordoir and Averill-Bates 2016), CBDV and CBG-treated cells were concomitantly incubated with the antioxidant NAC to assess if oxidative stress was associated with the observed effects. The caspase-9 activation induced by CBDV was significantly (*p* < 0.001) reversed by NAC, while this was not verified for caspase-8 (Fig. 3A, B). CBDV did not affect mitochondrial function, whereas CBG induced a decrease of Δψm that was dependent of ROS/RNS production (Fig. 3D), as the loss of Δψm was significantly (*p* < 0.01) reversed by NAC.

**Fig. 4:**
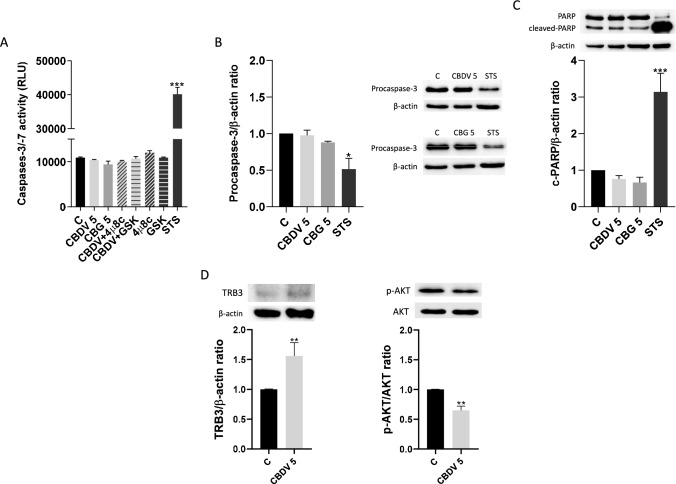
Analysis of the main biomarkers of apoptotic cell death and the expression of TRB3 and p-AKT proteins, for CBDV and CBG-treated cells. **A** Evaluation of the activity of effector caspases-3/-7, and of the expression of pro-caspase-3 **B** and cleaved-PARP **C** by Western blotting. **D** Evaluation of the protein expression of TRB3 and of AKT phosphorylation (p-AKT), by Western blotting. CBDV-treated cells were pre-incubated 4µ8c (1 µM) or GSK (0.5 µM), to analyze the role of UPR pathways in the activity of effector caspases. STS (10 µM) was used as inducer of apoptosis. The results are presented as mean ± SEM (n = 5). Significant differences between control and treatments are indicated by * (*p* < 0.05), ** (*p* < 0.01) and *** (*p* < 0.001)

To confirm the occurrence of apoptosis, the activation of the executor caspases-3/-7, as well as the proteolytic cleavage of PARP-1 protein were assessed in both cannabinoid-treated cells. Neither CBDV nor CBG induced the activation of the effector caspases-3/-7 at 48 h (Fig. 4A), which was confirmed through evaluation of the protein expression of pro-caspase-3 by western blotting (Fig. 4B). Moreover, the cannabinoids did not trigger PARP-1 cleavage, a hallmark of apoptosis (Fig. 4C).

To further understand the mechanisms behind CBDV effects, the expression of TRB3, inducible by ER stress and described as being involved in cell death by the ATF4/CHOP axis, was analyzed by Western blotting (Ohoka et al. 2005). In addition, as TRB3 has been reported to inhibit the activity of AKT protein kinases (Hosoi et al. 2007), the expression of AKT and p-AKT was also examined. CBDV significantly increased (*p* < 0.01) TRB3 expression, while the protein expression of phosphorylated AKT was decreased (*p* < 0.01) (Fig. 4D).

### Cannabidivarin and cannabigerol dysregulate the angiogenic activity of extravillous trophoblast cells

**Fig. 5:**
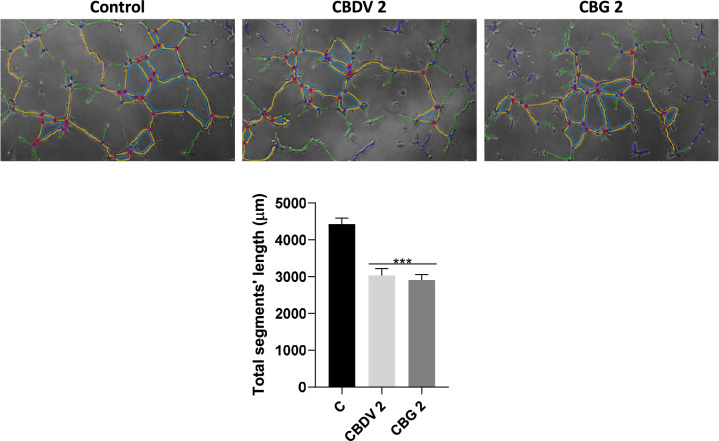
Impact of CBDV and CBG on tube formation by HTR-8/SVneo cells. Results are showed as the mean ± SEM of total segments’ length (µm) (n = 5). Legend: Red surrounded by blue—junctions; Segments—yellow; Branches—green; Meshes—cyan; Extremities—Orange; Artefactual branches—blue; Nodes—magenta. Significant differences between the control and CBDV and CBG are indicated by *** (*p* < 0.001)

**Fig. 6:**
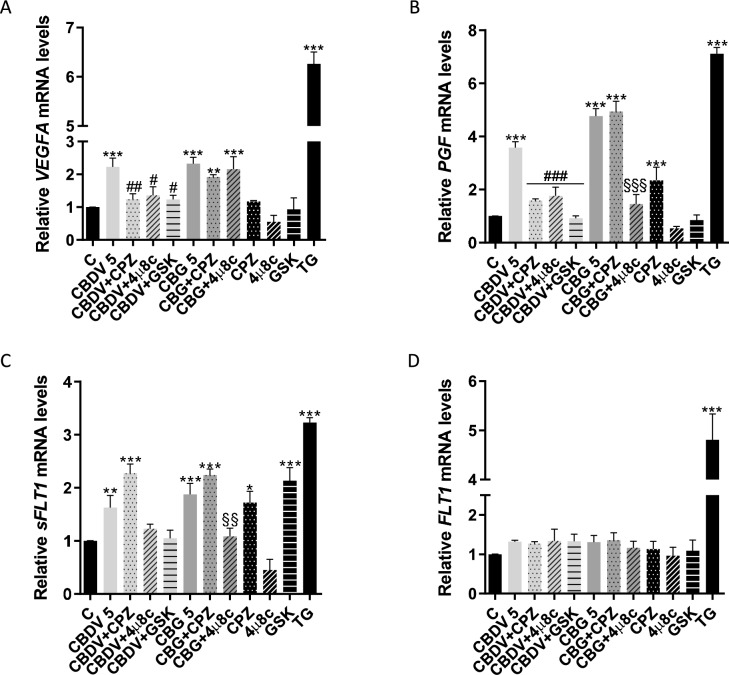
CBDV and CBG dysregulate the gene expression of angiogenesis-related factors. **(A-D)** Analysis of the mRNA levels of *VEGFA*, *PGF*, *sFLT1* and *FLT1*, evaluated through RT-PCR. To explore the involvement of TRPV1 and UPR pathways on the changes observed in CBDV and CBG-treated cells (5 µM), they were pre-incubated with CPZ (0.2 µM), 4µ8c (1 µM) or GSK (0.5 µM). TG (0.1 µM) was used as ER stress inducer. The results are presented as mean ± SEM (n = 5). Significant differences between control and treatments are indicated by * (*p* < 0.05), ** (*p* < 0.01) and *** (*p* < 0.001); between CBDV-treated cells with or without CPZ, 4µ8c or GSK are denoted by # (*p* < 0.05), ## (*p* < 0.01) and ### (*p* < 0.001); between CBG-treated cells with or without CPZ or 4µ8c are denoted by §§ (*p* < 0.01) and §§§ (*p* < 0.001)

Since ER stress is known to regulate angiogenesis (Binet and Sapieha 2015), which is fundamental for placentation, we also explored the impact of CBDV and CBG in HTR-8/SVneo endothelial-like behavior through the tube formation assay, which is currently used as a measure of in vitro angiogenesis. In this assay, a lower concentration (2 µM) was used as with higher concentrations the cells were not able to form the tube-like structures. Furthermore, the expression of several angiogenic factors of the VEGF family known to be involved in placental angiogenesis were also investigated. In addition, given the pro-angiogenic function of TRPV1 (Smani et al. 2018), CPZ was co-incubated with the cannabinoids to explore the role of this receptor. Both CBDV and CBG significantly (*p* < 0.001) decreased total segment length of the tubes (Fig. 5). Moreover, CBDV and CBG modified the mRNA levels of angiogenesis-related factors. Both compounds significantly (*p* < 0.001) increased gene expression of the pro-angiogenic factors *VEGFA* and *PGF*, being this effect significantly (*p* < 0.01 and *p* < 0.001, respectively) dependent of TRPV1 only for CBDV (Fig. 6A, B). Also, the expression of the anti-angiogenic factor *sFLT1* was increased for both cannabinoids (CBDV, *p* < 0.01; CBG, *p* < 0.001), in a TRPV1-independent manner (Fig. 6C). The mRNA levels of *FLT1* were not altered by any treatment (Fig. 6D).

### The role of ER stress on the effects induced by cannabidivarin and cannabigerol

To unveil the relevance of the activation of ER stress on the actions of CBDV and CBG, we used the inhibitors of IRE1 and PERK pathways, 4µ8c and GSK, respectively. The activation of caspases-8 and -9 caused by CBDV was significantly (*p* < 0.001) reversed in the presence of these inhibitors (Fig. 3A, B). A similar significant (*p* < 0.01) reversion was observed for CBDV-induced ROS/RNS production. On the contrary, co-incubation of CBG with 4µ8c, failed to prevent ROS/RNS increase (Fig. 3C) and Δψm loss (Fig. 3D). Moreover, the inhibition of the UPR signaling pathways had no influence on the activity of caspases-3/-7 (Fig. 4A).

Considering the effects on the expression of angiogenic factors, the increase of *VEGF* and *PGF* by CBDV was fully reversed by the inhibition of IRE1 (*p* < 0.05, *p* < 0.001) and PERK (*p* < 0.05,* p* < 0.001) (Fig. 6A, B), whereas for *sFLT1* only a strong tendency for reversion was observed (Fig. 6C), without statistical significance. In the case of CBG, only *PGF* and *sFLT1* increments were prevented by the co-incubation with 4µ8c (*p* < 0.001, *p* < 0.01) (Fig. 6B, C).

## Discussion

In this study, we report the effects of CBDV and CBG on the placental extravillous trophoblast HTR-8/SVneo cells. Our results show a decrease in cell viability and an anti-proliferative effect induced by both cannabinoids. We observed a cell cycle arrest at G_0_/G_1_ phase and a decrease of S phase, a behaviour that was already reported for CBG in glioblastoma (Lah et al. 2021) and mesothelioma (Colvin et al. 2022) cell lines. For CBDV, it was previously demonstrated that it impairs DNA synthesis in diverse human normal and cancer cells (Russo et al. 2021).

Exposure to CBDV and CBG induces ER stress and UPR. Quantitative PCR analysis demonstrated that *HSPA5*/BiP levels were threefold higher than the untreated cells and that these effects were dependent on TRPV1 activation. This receptor is a target for both phytocannabinoids (De Petrocellis et al. 2011) and is involved in processes of cell death (Fonseca et al. 2018), ER stress (Vestuto et al. 2022) and angiogenesis (Smani et al. 2018). Moreover, CBDV and CBG caused ROS/RNS generation also through TRPV1 activation. Indeed, the relevance of this receptor was already reported for CBD-induced oxidative stress (de la Harpe et al. 2022). In our study, CBDV and CBG exposure activated the IRE1 branch of the UPR. This is an important observation, as the IRE1 arm of UPR is essential for placental development and embryonic viability, as demonstrated by Iwawaki et al*.* in a model of IRE1 and XBP1 KO mouse (Iwawaki et al. 2009). They found that ER stress was enhanced by disruption of IRE1 and XBP1, but this effect was not pro-apoptotic. Concerning the PERK branch, we found that contrary to CBG, CBDV was able to increase eIF2α phosphorylation, ATF4 and CHOP expression, though this pathway did not lead to apoptosis. In fact, when homeostatic pathways fail to restore metabolic equilibrium, the ER stress can induce apoptosis in many cell types through activation of both mitochondrial-dependent and -independent apoptotic machineries (Szegezdi et al. 2006). Only CBDV caused an increase in the activity of the initiator caspases-8 and -9. This effect was not detected when TRPV1 was inhibited, suggesting a role for this receptor in the CBDV triggered-caspase-8/-9 activation. Interestingly, we also showed that PERK and IRE1 pathways are involved in the activation of these initiator caspases. Nevertheless, CBDV failed to finalize apoptosis, as confirmed by the analysis of the activity of the effector caspases-3/-7, as well as through the expression of procaspase-3 and c-PARP, since these apoptotic hallmarks were not altered after CBDV treatment. Contrary to caspase-9, the activation of caspase-8 by CBDV was not prevented by the antioxidant NAC, suggesting that ROS production is involved in the activation of the former, an effect that was already reported in the neuroepithelioma SK-N-MC cell line (Kim and Park 2003). Curiously, although CBG induced a decrease of the Δψm, this was not accompanied by an increase of caspase-9 activity. Similar observations were reported in astrocyte cell cultures during oxygen–glucose deprivation (Reichert et al. 2001). Considering the significant depolarization of Δψm caused by CBG, ROS may be the underlying cause since this effect was reversed by NAC. To further explore the role of ER stress in these processes, the inhibitors of PERK and IRE1 pathways were co-incubated with CBDV and CBG. We verified that CBDV-induced ROS production occurred through activation of both UPR pathways. In contrast, the ER stress induced by CBG was not linked to ROS/RNS production and Δψm loss.

Intriguingly, considering the activation of initiator caspases together with CHOP increase, it would be expected the occurrence of apoptotic cell death in CBDV-treated cells, though this was not observed. In fact, stimulation of ER stress pathways does not appear to underly the interruption of the apoptotic process since the inhibitors 4µ8c and GSK did not affect the activity of the effector caspases. One possible explanation is the activation of a mechanism that may hinder the finalization of apoptosis. In fact, it is known that *TRB3* is a gene regulated by CHOP and in mild or transient cases of ER stress, TRB3 protein can act through a negative feedback, blocking the action of CHOP and the activation of pro-apoptotic factors (Ohoka et al. 2005). Moreover, TRB3 promotes the translocation of pro-caspase-3 to the nucleus, thus preventing caspase-3 activation in the cytoplasm (Shimizu et al. 2012). Indeed, our results demonstrate that CBDV increased the expression of TRB3. On the other hand, its expression was associated to AKT inhibition by CBDV (Bromati et al. 2011), which may be associated to the activation of caspase-9 by this compound (Li et al. 2017), as well as to the observed cell cycle arrest at the G_0_/G_1_ phase (Vadlakonda et al. 2013). Interestingly, it was already reported that the reduction of AKT activation can have a harmful effect on placentation, leading to IUGR (Burton and Yung 2011).

The major phytocannabinoids CBD and THC are known to cause ER stress. The former induced a TRPV1-dependent effect, accompanied by increased oxidative stress, in breast cancer cell lines (de la Harpe et al. 2022), while the latter caused ER stress involving cannabinoid receptors activation and mitochondrial disfunction, in the BeWo trophoblast cell line (Lojpur et al. 2019). In this work, we show that the minor cannabinoids CBDV and CBG disturb ER homeostasis in HTR-8/SVneo cells. It is well known that ER stress is a relevant regulator of angiogenesis in the placenta, mainly due to the modulation of VEGF and PlGF expression (Burton et al. 2009; Ghosh et al. 2010; Iwawaki et al. 2009). In addition, when increased, it can lead to a pro-inflammatory state that modifies the behaviour of maternal endothelial cells, contributing to the onset of preeclampsia (Burton and Yung 2011). We recently demonstrated, using HTR-8/SVneo cells, the impact of CBD and THC in placental angiogenesis (Alves et al. 2023). Furthermore, THC is able to increase the mRNA levels of *VEGFA* and *FLT1*, through involvement of ER stress (Lojpur et al. 2019). Besides exploring the effects of CBDV and CBG on the formation of tube-like structures by the EVT model and on the gene expression of anti- and pro-angiogenic factors, we also wanted to clarify the role of ER stress in these events. Both cannabinoids affected tube-like structures formation, causing a decrease of their total segment length. In addition, our results revealed an evident impact on the expression of pro- and anti-angiogenic factors. In fact, these compounds were able to increase the transcription of the two pro-angiogenic factors *VEGFA* and *PGF*. However, while CBDV induced this effect by the activation of the UPR pathways IRE1 and PERK, CBG acted through the IRE1 arm, which had an impact in the *PGF* but not in the *VEGFA* expression. A study indicates that *PGF* is transcriptionally regulated by ATF4, but contrary to our results for CBDV, that led to a decreased expression and secretion of PlGF (Mizuuchi et al. 2016). Nonetheless, an increase of the mRNA levels of *PGF* was already associated to IRE1 activation in the hepatocellular carcinoma HepG2 cell line (Vandewynckel et al. 2016), which matches with our results for both cannabinoids. On the other hand, the increase of *VEGFA* promoted by CBDV through UPR activation is in accordance with the literature (Ghosh et al. 2010). In fact, VEGF-A is elevated by ER stress (Abcouwer et al. 2002; Ferrara et al. 1996). Nevertheless, the actions of both cannabinoids were not sufficient to cause alterations in the expression of *FLT1* that codes for the membrane receptor VEGFR1 but caused an increase in *sFLT1* expression. This anti-angiogenic factor is a soluble receptor that binds to free VEGF and PlGF, thus reducing their bioavailability for the membrane receptor and, in that way, preventing their effects. Thus, the observed increase in *sFLT1* expression may also be a response to the increment in the pro-angiogenic factors *VEGFA* and *PGF*, or even act as a regulator of other players in angiogenesis (Failla et al. 2018). As with the pro-angiogenic factors, the IRE1 pathway of the UPR seems to be involved in the effect induced by CBG, since 4µ8c prevented the transcription of *sFLT1*. Nevertheless, we verified the same pattern for CBDV with PERK and IRE1, though this did not have a statistically significant outcome.

In conclusion, CBDV and CBG induced ER stress in the HTR-8/SVneo cells, with subsequent UPR activation, but acting through different mechanisms. CBDV treatment was associated with an increase in CHOP expression, though cells were able to neutralize the finalization of the apoptotic process, possibly through the negative feedback of TRB3. On the other hand, CBG seemed to induce a milder ER stress, with the involvement of the IRE1 pathway, without triggering apoptosis. Nevertheless, ER stress induced by the minor phytocannabinoids affect the angiogenic process and the proliferation of trophoblastic cells, which are crucial for the development of placenta. To the best of our knowledge, this is the first time that these effects are described for CBDV and CBG in placenta cells. Therefore, with this work we expect to contribute to the understanding of the negative impact of these less studied minor phytocannabinoids when consumed during such a critical phase of pregnancy development.

## Data Availability

All data generated or analyzed during this study are included in this published article.
